# Microbiological Assessment of Titanium Plates Coated with PLGA, Chitosan, and/or Meropenem: An In Vitro Study

**DOI:** 10.3390/antibiotics11111565

**Published:** 2022-11-06

**Authors:** Mohammad Al-Qubaisey, Rita Khounganian, Abdulhakim Al-Badah

**Affiliations:** 1 Department of Dentistry, Riyadh 2nd Health Cluster, P.O. Box 60169, Riyadh 11545, Saudi Arabia; 2 Department of Oral Medicine and Diagnostic Sciences, College of Dentistry, King Saud University, P.O. Box 60169, Riyadh 11545, Saudi Arabia; 3 Microbiology Laboratory, College of Dentistry, King Saud University, P.O. Box 60169, Riyadh 11545, Saudi Arabia

**Keywords:** titanium plates, confocal microscopy, zone of inhibition, colony-forming unit, chitosan, PLGA, meropenem, *Staphylococcus aureus*, *Pseudomonas aeruginosa*

## Abstract

This investigation was conducted to evaluate the efficacy of coated titanium plates against gram-positive *Staphylococcus aureus* (*SA*) and gram-negative *Pseudomonas aeruginosa* (*PA*) using various methods. The Colony-forming unit “CFU” was higher in chitosan (CH) in relation to *PA* than in poly lactic-co-glycolic acid (PLGA) in relation to *SA*, followed by meropenem-chitosan “MC” coated plates in relation to *PA* and *SA*. A significant difference in the zone of inhibition (ZOI) of *SA* was determined in MP, MC, and meropenem (MEM). *PA* was significantly inhibited by MP, MEM, then MC, and the largest ZOI among *SA* and *PA* groups were MP coating. Using an MTT assay, MP had the lowest bacterial viability in the *SA* group, followed by MC and MEM, with no statistically significant difference between the PLGA or CH alone nor the polymers augmented with MEM. Using confocal microscopy, MP-coated plates were seen to have the highest bacterial inhibition, followed by MC, MEM, PLGA, and CH. In the *PA* group, MP had the highest bacterial inhibition, followed by MEM, MC, CH, and PLGA. The uncoated group presented the lowest inhibition in relation to both *SA and PA*. Conclusively, coating titanium plates with PLGA or CH with MEM appeared to enhance the antibacterial efficacy as opposed to MEM without polymers through bacterial adhesion inhibition, hindering biofilm formation and preventing bacterial proliferation.

## 1. Introduction

Michelet and Festal suggested using microplates to treat facial fractures in 1972. In the late 1970s, Harle and his coworkers popularized the use of microplates in orthognathic surgery [1]. Initially, these plates were made from metal alloys or stainless steel. Titanium has replaced stainless steel and other metal alloys because of their lack of corrosion resistance and poor cytocompatibility [2].

However, titanium has several drawbacks, including toxicity and potential hypersensitivity. According to current estimates, 10–12% of plates need to be removed because of infection, exposure, discomfort, or pain [3]. According to another study, titanium plates and screws had a 10% failure rate. Patients with titanium plates complained for various reasons, even two decades after surgery [4].

Conventional approaches for delivering small molecule medications to other infection sites are challenging-to-treat infections caused by microplates [5]. Because unbound biomacromolecules frequently have a short half-life and a propensity to degrade and denature in physiological settings, recent advances in bio-macromolecular treatments have increased the necessity of creative drug delivery techniques [6]. Polymeric nanoparticles, particularly those created with biodegradable polymers, are excellent carriers of macromolecular medicines, given their advantages over traditional drug delivery systems [7].

This study intends to confirm and examine the use of poly lactic-co-glycolic acid (PLGA), or chitosan (CH), supplemented with meropenem (MEM), as a coating for titanium plates to prevent bacterial adherence and colonization. As far as we know, no studies have been published on this aspect. In routine oral and maxillofacial treatments, especially those involving reconstruction when the patient might be medically compromised, these modified titanium plates are meant to take the place of their present uncoated counterparts as a preventive against infection.

PLGA is one of the most used polymers for biological applications and drug administration. It is a copolymer of lactic acid and glycolic acid that has been demonstrated to be biocompatible, biodegradable, and beneficial for drug release over a more extended period than prior polymers. It is hydrolyzed inside the body to produce biodegradable metabolites, primarily lactic acid and glycolic acid [8]. CH is derived from the shells of marine crustaceans and is one of the substrates with the highest production and biodegradability rates in nature. CH provides several benefits in biomedical applications, including biocompatibility and regulated biodegradability, which confer nontoxic and noninflammatory qualities on its degradation products. Because of the variety of CH applications, several shapes and forms are available. Because of its solubility in water, it can form films and scaffolds [9]. Meropenem “MEM” belongs to the carbapenem subclass of antibiotics. It possesses a broad spectrum of activity because it has the broadest spectrum among the β-lactams group [10].

The goal of coating titanium plates with polymeric carriers loaded with antimicrobial agents is to lower the likelihood of infection, thus reducing the chance that patients will require subsequent surgeries. *Staphylococcus aureus* (*SA*) is classified as a gram-positive coccus. *SA* is a major cause of nosocomial and community-acquired infection and represents a significant burden on the health care system; it has long been linked to various infectious diseases ranging from skin infections, abscesses, and wound infections to more severe infections such as pneumonia, meningitis, osteomyelitis, and endocarditis [11,12]. *Pseudomonas aeruginosa* (*PA*) is a gram-negative, rod-shaped, saccharolytic, non-spore-forming, aerobic bacillus with polar flagella used for motility. *PA* is a widespread bacterium found in soil, water, plants, and animals [13]. *PA* can develop biofilms on implanted devices. Localized infections can be complicated to diagnose with routine clinical microbiology and frequently fail to heal despite intensive antibiotic therapy [14].

Over the past decades, many assays for bacterial viability and quantification have been proposed, such as the 3-(4,5-dimethylthiazol-2-yl)-2,5-diphenyl-2H-tetrazolium bromide “MTT” assay [15,16,17]. It was utilized since it is considered the gold standard for cytotoxicity and cell proliferation assessment [17].

The present investigation was conducted to assess the antimicrobial effect of titanium plates coated with PLGA, CH, and/or MEM.

## 2. Materials and Methods

### 2.1. Samples Preparation

The present study was carried out on 96 single-hole segments of 0.4 mm-thick, low-profile MatrixMIDFACE titanium plates (DePuy Synthes, Raynham, MA, USA). Coating materials were divided into two categories:

A. Carriers: composed of copolymers, poly lactic-co-glycolic acid “PLGA” and chitosan “CH”.

B. Antimicrobial agent: meropennem “MEM”.

The following coating materials—PLGA or CH and MEM—were investigated using 1% *w*/*v* in acetone (Sigma-Aldrich, St. Louis, MO, USA) of each solution. They were sprayed onto the entire surface of the plates using an airbrush system (Harder & Steenbeck, Oststeinbek, Germany) [18]. The plates were left to air dry overnight at room temperature in the safety cabinet. The coating procedure was carried out accordingly:

Solution 1: 25 mg poly lactic-co-glycolic acid “PLGA” and 5 mg meropenem “MEM” in 2.5 mL acetone “MP”.
Solution 2: 25 mg chitosan “CH” and 5 mg meropenem “MEM” in 2.5 mL acetone “MC”.
Solution 3: 5 mg meropenem in 2.5 mL acetone “MEM”.
Solution 4: 25 mg poly lactic-co-glycolic acid in 2.5 mL acetone “PLGA”.
Solution 5: 25 mg chitosan in 2.5 mL acetone “CH”.

Each plate was weighed before and after spraying to ensure the final modified plates contained 5 mg of MEM and 25 mg of the polymer [19].

### 2.2. Bacteria Strains

Gram-positive aerobic (*SA* ATCC^®^ #25923^TM^) and gram-negative aerobic (*PA* ATCC^®^# 27853 ^TM^) bacterial strains were selected for this research. Bacterial adhesion, proliferation, growth, and biofilm formation were accordingly studied.

### 2.3. Colony-Forming Unit (CFU) Assay

Working surfaces, equipment, and tools were sterilized following the Occupational Safety and Health Administration’s guidelines [20].

(A) Media Preparation

Tryptic soy broth (TSB) was prepared according to the manufacturer guidelines (Sigma-Aldrich, USA) in capped 5 mL glass tubes, then sterilized in the autoclave at 121 °C and 15 psi for 20 min. Similarly, tryptic soy agar (TSA) was prepared, poured into a sterile 100 mm Petri dish under aseptic conditions inside the safety cabinet, then covered with a lid and left overnight to solidify. All the media were stored at 4 °C until needed [20].

(B) Bacteria Cultivation

TSB was used to cultivate the two types of bacteria (*SA* and *PA*). The cultivation took place overnight in an incubator set at 37 °C. The following day, the culture was diluted to 1:50 in fresh TSB and incubated for 3 h at 37 °C to achieve the logarithmic growth phase.

A total of 25 μL (equal to 50 McFarland) of the bacteria culture was injected into 18 wells on six lines of a flat-bottomed 96-well microtiter polystyrene plate. One plate was used for each type of bacteria. One segment of each coated plate was placed into the 18 wells with the bacteria solution, including the uncoated negative control group. The polystyrene plate was gently spun at 20 RPM for 24 h and kept at 37 °C to generate biofilms. Each titanium plate segment was removed using sterile forceps, gently washed with phosphate-buffered saline (PBS) to remove planktonic bacterial cells, then placed into Eppendorf^®^ Safe-Lock microcentrifuge tubes (Sigma-Aldrich, USA) that were labeled with each coating material name, then 200 μL of TSB was added and vortexed at 500 rpm for 10 s to dislodge the bacterial biofilm from the titanium plates [21,22].

(C) Serial Dilution

**Figure 1:**
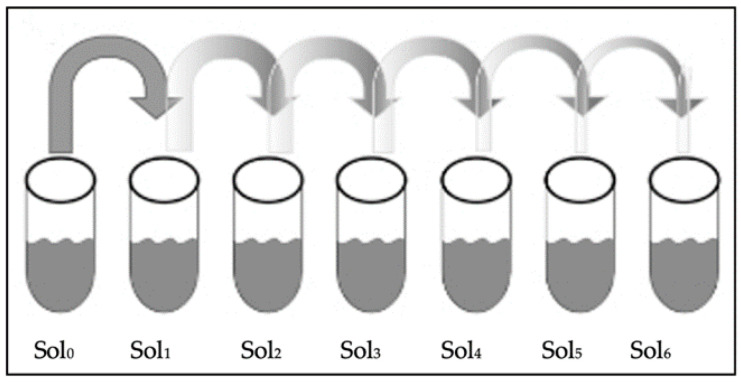
Serial dilution stock of bacteria (Sol _0_) up to the sixth dilution (Sol _6_).

The test tubes with TSB were labeled 1–6 for the serial dilution of the bacteria solution. A total of 10 μL of the bacterial solution from the Eppendorf^®^ tubes (Solution 0) was placed into the test tubes with TSB. A total of 1 mL of solution 0 was placed into the test tube labeled 1, then vortexed at 500 rpm for 10 s. The serial dilution was carried out as follows: 1 mL was removed from test tube 1, added to test tube 2, and then vortexed. The process was repeated for the remaining test tubes, as shown in Figure 1 [23].

(D) Streaking

A total of 100 µL of the diluted culture was placed onto the Petri dish, which was labeled with the dilution number. Using a sterile, disposable spreading loop, the culture was evenly spread with a zigzag gliding motion, the plate was rotated 180°, and the zigzag motions were repeated for each dilution. All the agar dishes were incubated at 37 °C overnight [21].

(E) CFU Counting

One Petri dish for each group of the corresponding coating materials was selected based on the ability to manually count the individual colonies, keeping the dilution factor constant for each group. The resulting number was multiplied by 10 to the power of the dilution factor [21].

### 2.4. Bacterial Zone of Inhibition (ZOI)

Bacterial inhibition zone diameter tests were assessed in accordance with the Kirby–Bauer method, as replicated by previous investigators [17,18,19,20]. The TSA media was prepared similarly to step 2.3 A.

A thin film of bacteria was applied using a 10 μL bacteria solution on a Petri dish, then streaked using a sterile, disposable loop with a zigzag gliding motion. The plate was rotated 180°, and the zigzag motions were repeated. This was done for a total of 12 plates (six for each type of bacteria).

A single segment of the coated and uncoated titanium plates (similar to step 2.1) was placed in the center of each Petri dish and labeled accordingly (*PA* or *SA* for the bacteria, and MEM, PLGA, CH, MP, MC, or uncoated), and the agar dishes were incubated at 37 °C overnight. The following day, the ZOI surrounding each titanium segment was measured using a ruler placed over the Petri dish, and the value in millimeters was recorded in a table [24].

### 2.5. MTT Assay

An MTT assay was employed to evaluate microorganism proliferation and growth over the coated and uncoated titanium plates. The bacteria cultivation was done similarly to step 2.3 B.

A total of 25 μL of the dissolved biofilm was taken and injected into a new flat-bottomed 96-well microtiter polystyrene plate, per the MTT kit manufacturer’s instructions (Sigma-Aldrich, USA). A total of 10 μL of MTT labeling reagent was injected into each well and incubated at 37 °C for 4 h, then 100 μL of stabilization buffer was added and incubated at 37 °C overnight. The following day, the plate was read using a Synergy HT microplate reader (Biotek, Winooski, VT, USA). The wavelength to measure the absorbance of the formazan was set at 550 nm [25].

After obtaining the results from the Biotek Gen5 Version 2.00 software, they were exported to Microsoft Excel^®^ (Microsoft 2022, USA), and the bacteria viability was calculated using the following formula [26]:

$$\frac{Sample-mean\;of-ve\;control}{mean\;of+ve\;control}\times 100$$

### 2.6. Confocal Microscopy Assessment

The biofilm was indirectly measured by staining the bacterial biomass with a live and dead fluorescent dye (Biotium, Fremont, CA, USA) [22]. As previously described, the coated and uncoated plates were incubated with the bacterial suspension, and the bacteria biofilm was suspended in TSB. The dye stock solutions were prepared per the manufacturer’s instructions (Biotium, USA). The bacterial cells were harvested by centrifugation of Eppendorf^®^ tubes at 10,000 RPM for 5 min in a microcentrifuge (Sigma-Aldrich, USA). A total of 1 μL of the dye mixture was added to 100 μL of the bacterial suspension, then gently mixed and kept in darkness for 15 min at room temperature.

A total of 5 μL of the sample was transferred to a glass slide and cover-slipped. The slides were viewed using the confocal microscope C2 CLM (Nikon, Tokyo, Japan) using a FITC filter. The images were captured using NIS Elements software (Nikon, Japan). Then the number of live or dead bacteria was calculated using ImageJ software (version 1.53, Rockville, MD, USA). The live bacteria percentage was calculated by dividing live bacteria by the dead bacteria count, then multiplying by 100 [18,22].

### 2.7. Statistical Analysis

The collected data were analyzed using the Statistical Package for the Social Sciences software version 26.0 (IBM Inc., Chicago, IL, USA). Descriptive statistics (mean and standard deviation) were used to express all quantitative variables. Quantitative data were obtained from the CFU calculation of samples from all groups. One-way ANOVA was used to compare the data obtained from the ZOI analysis of samples from all groups within the two types of bacteria, and a t-test was used to evaluate the overall difference between and within the materials among the two types of bacteria.

A Kruskal–Wallis nonparametric test was performed to compare the quantitative data obtained from the MTT analysis of samples because normality was not satisfied. One examiner carried out all assessments and repeated them three times to confirm reproducibility and reliability. The results were considered statistically significant when *p* ≤ 0.05.

## 3. Results

### 3.1. CFUs

Overall, the maximum number of CFUs detected was related to the uncoated titanium plates (-ve control), with the *PA* followed by *SA*, resulting in 327 × 10^4^ and 164 × 10^4^, respectively. As for the coated counterparts, the maximum number of CFUs was related to the PLGA-coated plates, with *PA* yielding 83 × 10^4^, followed by the CH-coated plates related to *SA*, resulting in 30 × 10^4^.

**Figure 2:**
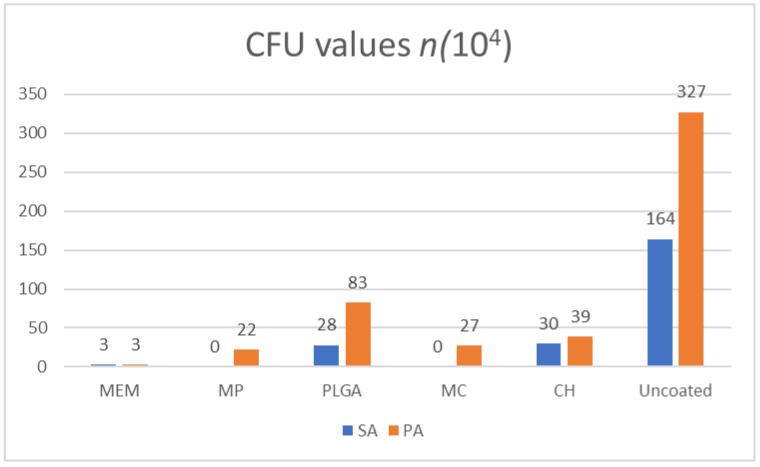
CFU count for *SA* and *PA* against the tested materials.

The values of CH-coated plates in relation to *PA* were 39 × 10^4^, whereas PLGA-coated plates related to *SA* were 28 × 10^4^, followed by MC-coated plates in relation to *PA* and *SA*, yielding 27 × 10^4^ and zero, respectively. In contrast, MP in relation to *PA* and *SA* resulted in 22 × 10^4^ and zero, respectively. As for the MEM-coated plates, *PA* and *SA* were equal, resulting in 3 × 10^4^, as shown in Figure 2.

### 3.2. Bacterial ZOI

**Figure 3:**
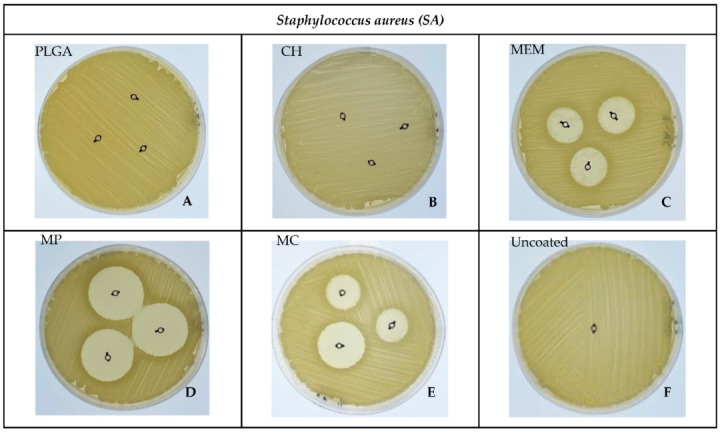
Showing the inhibition zones among the different materials against *SA*.

**Figure 4:**
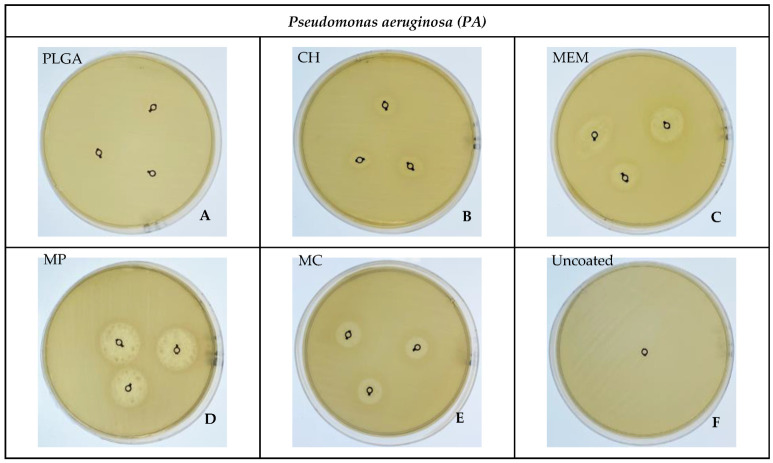
Showing the inhibition zones among the different materials against *PA*.

A significant difference in inhibition of *SA* was determined in MP, then MC, followed closely by MEM, as shown in Figure 3C–E. In contrast, *PA* was significantly inhibited by MP, followed to a lesser extent by MEM, then MC, as shown in Figure 4C–E, compared to the absence of a ZOI in the PLGA, CH, and -ve control uncoated groups related to *SA* and PLGA in addition to the -ve control group for *PA,* as shown in Figure 3A,B,F and Figure 4A,F. Overall, the largest inhibition zone among *SA* and *PA* groups was related to the MP coating, resulting in 29.67 ± 0.58 mm and 18.33 ± 1.15 mm, respectively, as shown in Table 1, Figure 3D and Figure 4D.

### 3.3. MTT

The highest bacteria viability was observed in the uncoated plate groups in relation to *SA* and *PA* (58% and 95%, respectively), whereas the lowest was observed in the *SA* group in relation to CH and PLGA, yielding 2.47% and 2.60%, respectively, followed by MP and MC at 3.36% and 3.69%, respectively, with no statistically significant difference between the groups coated with PLGA or CH alone, nor the polymers augmented with MEM (MP or MC). The *p*-value was >0.05. The MEM-coated plates had a statistically significant difference compared to the PLGA, CH, and MP groups related to *SA*, where the *p*-value was <0.05.

In the *PA* group, the lowest bacteria viability was detected in relation to PLGA and MP coating, resulting in 1.58% and 1.90%, respectively, with no statistically significant difference between PLGA, CH, MP, and MC (*p*-value was >0.05), as shown in Table 2. However, there was an overall significant difference among the different materials regarding *SA* and *PA* (*p* = 0.000).

Regarding the MTT differences within each material for *SA* and *PA,* no apparent statistically significant differences were noted for PLGA, CH, or MC, whereas statistically significant differences were observed in the MP, MEM, and uncoated groups, as shown in Table 3.

### 3.4. Confocal Microscope Imaging

**Figure 5:**
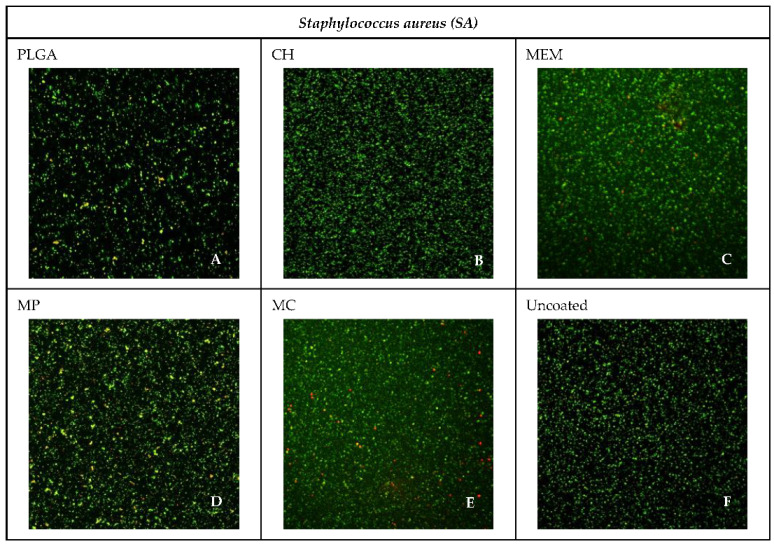
Confocal microscope images of the different materials for *SA*.

**Figure 6:**
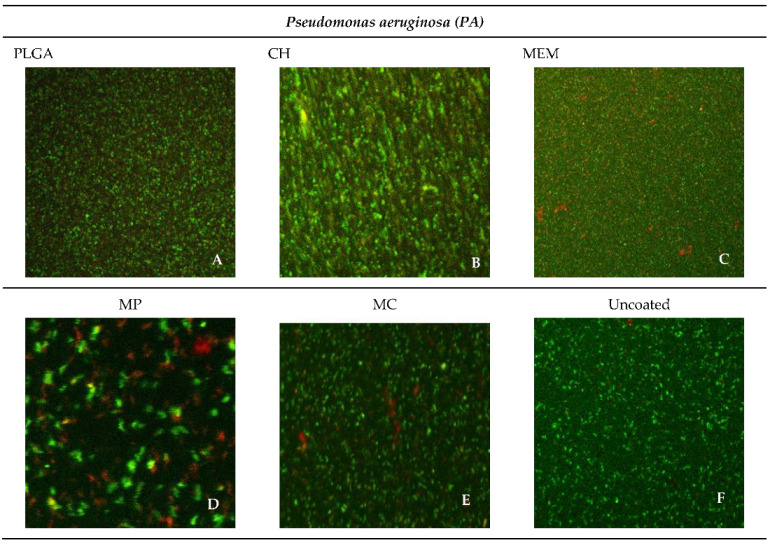
Confocal microscope images of the different materials for *PA*.

The most abundant live bacteria were found in the negative control group of both *SA* and *PA*, whereas the lowest bacteria viability was found in the MP group related to *SA* 42.63% and PA 38.79%, followed closely by MEM related to *PA* 40.70%, as shown in Figure 5 and Figure 6.

Overall, the frequency percentage of the lowest inhibition of the most viable bacteria was noticed among the CH and PLGA, followed by MEM, MC, and MP in relation to *SA*. In contrast, PLGA had the lowest inhibition compared to CH, MC, MEM, and MP in relation to *PA*. The uncoated group presented the lowermost inhibition in both SA and PA groups, as shown in Table 4.

A correlation between the various methods used to investigate the antibacterial efficacy, including the CFU, ZOI, MTT assay, and confocal imaging, was cross-tabulated for direct comparison, as shown in Table 5.

## 4. Discussion

Successful identification, isolation, culturing, and characterization of microorganisms depend on the capacity to accurately estimate the bacterial concentration. Given their ubiquity, propensity for exponential growth, and unique physiological requirements, prokaryotes may be challenging to quantify. This problem is made more difficult by the four-phase process by which bacteria multiply (lag, log, stationary, and death) [27].

Consequently, the present baseline in vitro experiment was conducted to quantify the bacteria adherent to the coated titanium plates, evaluating the efficacy of their potential antibacterial effects using CFUs, ZOIs, MTTs, and confocal microscope imaging.

Various procedures were used to quantify bacteria accurately by serial dilution. This technique was first described over a century ago by Robert Koch. Since then, it has become the gold standard for quantifying bacteria [23,27]. It is one of the most straightforward techniques for attaining manageable concentrations of certain bacteria, and it is accompanied by Petri dish streaking. Each colony on the dish is counted to estimate the number of CFUs per milliliter in a given solution [20].

Having a locally present delayed-release antibiotic to hinder bacterial colonization on the titanium plate surface might be the solution to reduce and possibly eliminate the need for further surgical intervention to remove the source of infection [12]. Because the bacteria within the biofilm are more resistant to treatment with antimicrobial agents than their planktonic counterparts, routine antibiotic treatments are usually ineffective at reducing infection. To date, there are no reasonable means to irradicate the infection after it has occurred, and hardware removal is often the best way to eliminate the problem [12,28,29].

Counting the resulting colonies will give insight into how the bacterial cells are affected by the various antibacterial agents subjected to in the initial steps of the experiment (the soft agar colony formation assay). In general, the maximum CFU detected was found to be related to the uncoated titanium plates (-ve control) with *PA* followed by *SA*; this is a typical result given the lack of inherent antibacterial properties of the titanium alloy and the virulence of *PA* compared to *SA*, resulting in the highest CFU among all the groups [6,11,30]. As for the polymer-coated plates, the resulting CFU was the second-highest count compared to the negative control group, which resulted in minimal bacterial inhibition because of the weak antimicrobial potentials of both PLGA and CH alone [18,31,32].

The plates coated with MC had reasonably lower CFU values than their polymer-only counterparts; similar results were obtained from the MP group. The MEM group resulted in a substantial overall inhibition for both *SA* and *PA*; this could be attributed to the lack of the polymer layer—thus, the absence of the delayed drug release—subjecting the bacteria to an immediate antibacterial effect in accordance with previous investigations [33,34].

The Kirby–Bauer technique, also known as the disk diffusion sensitivity test, involves testing several medications on a thin layer of bacteria streaked on a Petri dish with solid media. A ZOI is the resulting circular area surrounding the spot of the disk in which the bacterial growth is suppressed. Bhargav et al. reported that a ZOI is frequently used to measure the susceptibility of certain bacteria toward an antibiotic [24].

A statistically significant difference occurred in *SA* toward MP, MC, and MEM, respectively; this can be attributed to the delayed MEM release from the polymer layer because the ZOI experiment was carried out over 24–48 h. Similar behavior was observed in the *PA* group. However, the results were MP, MEM, and MC, respectively; this is ascribed to the different responses of *PA* and *SA* toward the polymers augmented with MEM, consistent with the research of Guillaume et al. [18,19]. The absence of a ZOI in the negative control group of both *SA* and *PA* is typical, given the lack of antibiotics [6,11].

Over the past years, many assays for bacterial viability have been proposed [21]; the tetrazolium salt reduction assay (MTT) is one of the well-known methods. It was chosen to assess and quantify bacterial adhesion on the titanium plate surfaces [25]. The MTT assay was additionally used to complement the previous tests for the quantitative assessment of the bacterial viability on the coated titanium plates. In this method, the yellow tetrazolium dye is reduced by the viable bacteria into an insoluble formazan precipitate that accumulates within only metabolically active cells [35]. This evaluation could be beneficial as a prediction of bacterial survivability.

The highest bacterial viability was noticed in the uncoated plates (i.e., they had the lowest antimicrobial effect). This is a normal reaction because this group does not contain an antibacterial agent (negative control). The lowest bacteria viability was observed in the *SA* group, related to CH and PLGA, followed by the MP and MC, where no statistically significant differences were noted. In contrast, the MEM-coated plates had a higher statistically significant difference than the PLGA, CH, and MP groups related to *SA*. In the *PA* group, the lowest bacteria viability was found in the PLGA and MP coating, with no statistically significant difference between PLGA, CH, MP, and MC. However, there was a significant difference among the different materials regarding *SA* and *PA*.

According to Goy et al.’s interpretation, this could be because of the polymer’s ability to potentially prevent the development of biofilms and long-term microbial adhesion and colonization [32]. Additionally, the polymers provide excellent penetration into the deeper layer of the biofilm, eradicating the bacteria because of their high drug-loading efficiencies, hydrophilic surface, negative charge, and electrostatic contact, in addition to the differences in structure and characteristics of *SA* and *PA* [32,33,36,37].

The fundamental purposes of the confocal microscope are to generate a point source of light and reject out-of-focus light, which enables the imaging of deep tissues with high resolution and the optical sectioning of imaged samples for 3D reconstruction. For the localization of proteins and structures in whole cells and tissues and to observe the rapid dynamics of living cells, confocal imaging with fluorescent probes, dyes, and the vast array of accessible secondary antibodies have been employed to great advantage [38,39].

The highest percentage of live bacteria was found in the negative control group of both *SA* and *PA,* whereas the lowest bacteria viability was found in the MP group related to *SA* and *PA*, followed closely by MEM related to *PA*. Overall, the percentage of the lowest inhibition (the most viable bacteria) was noticed among CH and PLGA, followed by MEM, MC, and MP in relation to *SA,* whereas PLGA had the lowest inhibition when compared to CH, MC, MEM, and MP in relation to *PA*. The uncoated group presented the lowest inhibition in both *SA* and *PA*.

Within the frame of the present findings, the direct deposition of MEM with or without polymers on the titanium plate’s surface inhibited bacterial colonization. The results obtained from the various methods used to investigate the antibacterial efficacy in the present study, including the CFU, ZOI, the MTT assay, and confocal imaging, complemented and supported each other. The uncoated group presented overall the worst antimicrobial performance. Regarding the behavior of *Staphylococcus aureus*, CFU values were almost similar in PLGA and CH, followed by MEM, MP, and MC. The largest ZOI was found to be related to MP, followed closely by MC and MEM, with the absence of an inhibition zone in the PLGA group. The percentage of live bacteria, according to the MTT test, from the second highest to lowest, was MEM, MC, MP, PLGA, and CH. In comparison, the second-highest live bacteria per the confocal microscope were found to be MP, PLGA, CH, MEM, and MC.

Concerning the behavior of *Pseudomonas aeruginosa*, the CFU values from the second highest to lowest were PLGA, CH, MC, MP, and MEM. The largest ZOI was found to be related to MP, MEM, MC, CH, and PLGA. The percentage of live bacteria per the MTT test from the second highest to the lowest was MEM, MC, CH, MP, and PLGA. In contrast, the second-highest live bacteria per the confocal microscope were found to be PLGA, MP, MEM, CH, and MC.

Despite the resulting differences among some of the tests, we can conclude that MC had the best overall balanced performance between drug release and antimicrobial properties in relation to SA bacteria, while MP was the best regarding PA, as presented in Table 5.

These findings, if clinically applicable, could decrease the chances of infection. Therefore, if a biofilm develops on the plate’s surface, the therapeutic agent’s presence at the plate site can immediately increase the antibacterial efficacy.

To date, there are no coated titanium plates with antibacterial properties for use in oral and maxillofacial surgery on the market. Furthermore, active ingredients, such as antibiotics, have a limited shelf life and sensitivity to storage conditions. This might complicate the manufacturing process, thus delaying the time to commercialization and increasing the expense. This can be supported in the future by a complementary in vivo experimental investigation in animals, followed by a randomized clinical trial “RCT” to support the results of the present baseline research and aid in a market feasibility study for future commercial-scale manufacturing.

## 5. Conclusions

Coating titanium plates with PLGA or CH with MEM appeared to enhance antibacterial efficacy as opposed to MEM without polymers through bacterial adhesion inhibition, hindering biofilm formation and preventing bacterial proliferation. Surface treatment of titanium plates appears to be a challenging, versatile approach to avoid the development of infections. Further studies are needed to develop new bio-functionalization methods to enhance the antibacterial efficacy of the titanium plates by modulating the polymers and the antibiotic release profile.

## Figures and Tables

**Table 1:** Zone of inhibition among the different materials for *SA* and *PA*.

Bacteria	Material	Mean ± SD	Median	Range	Mean Rank	K-W *p*-Value **
***SA***	PLGA	0.00	0.00	0.00	5.00	0.006 *
	MP	29.67 ± 0.58	30.00	1.00	17.00	
	CH	0.00	0.00	0.00	5.00	
	MC	21 ± 1.73	20.00	3.00	13.00	
	MEM	20 ± 1.0	20.00	2.00	12.00	
	Uncoated	0.00	0.00	0.00	5.00	
***PA***	PLGA	0.00	0.00	0.00	3.50	0.005 *
	MP	18.33 ± 1.15	19.00	2.00	17.00	
	CH	8.33 ± 1.15	9.00	2.00	8.00	
	MC	13.33 ± 1.15	14.00	2.00	11.67	
	MEM	14.33 ± 0.58	14.00	1.00	13.33	
	Uncoated	0.00	0.00	0.00	3.50	

* Statistically significant at *p* ≤ 0.05. ** Kruskal–Wallis *p* value.

**Table 2:** MTT differences for *SA* and *PA* between materials.

Bacteria	Materials	Mean ± SD	*p*-Value	95% Confidence Interval		Multiple Comparison Test					
				Lower Bound	Upper Bound	PLGA	MP	CH	MC	MEM	Uncoated
***SA***	PLGA	2.60 ± 0.49	0.000 *	1.38	3.83	1					
	MP	3.36 ± 0.21		2.84	3.88	0.456	1				
	CH	2.47 ± 0.42		1.43	3.51	1.000	0.252	1			
	MC	3.69 ± 0.38		2.74	4.64	0.264	0.892	0.144	1		
	MEM	14.03 ± 0.65		12.41	15.64	0.000 *	0.003 *	0.000 *	0.144	1	
	Uncoated	57.99 ± 0.74		56.16	59.82	0.000 *	0.000 *	0.000 *	0.001 *	0.000	1
***PA***	PLGA	1.58 ± 0.11	0.000 *	1.30	1.85	1					
	MP	1.90 ± 0.23		1.32	2.47	0.544	1				
	CH	2.71 ± 0.21		2.18	3.25	0.024	0.080	1			
	MC	3.47 ± 0.26		2.82	4.12	0.013	0.012	0.133	1		
	MEM	24.24 ± 0.23		23.67	24.82	0.000 *	0.000 *	0.000 *	0.000 *	1	
	Uncoated	94.09 ± 0.23		93.51	94.67	0.000 *	0.000 *	0.000 *	0.000 *	0.000 *	1

* Statistically significant at *p* ≤ 0.05.

**Table 3:** MTT differences for *SA* and *PA* within each material.

Materials	Bacteria	Mean ± SD	*p*-Value
PLGA	*SA*	2.60 ± 0.49	0.062
	*PA*	1.58 ± 0.11	
MP	*SA*	3.36 ± 0.21	0.001 *
	*PA*	1.90 ± 0.23	
CH	*SA*	2.47 ± 0.42	0.452
	*PA*	2.71 ± 0.21	
MC	*SA*	3.69 ± 0.38	0.462
	*PA*	3.47 ± 0.26	
MEM	*SA*	14.03 ± 0.65	0.000 *
	*PA*	24.24 ± 0.23	
Uncoated	*SA*	57.99 ± 0.74	0.000 *
	*PA*	94.09 ± 0.23	

* Statistically significant at *p* ≤ 0.05.

**Table 4:** Showing the Percentages of live bacteria among the different materials against *SA* and *PA* using the confocal microscope.

Material	*SA*%	*PA*%
PLGA	94.10	93.73
CH	97.26	83.28
MEM	63.79	40.70
MP	42.63	38.79
MC	46.61	46.61
Uncoated	99.74	99.43

**Table 5:** Correlation between the different test methods against the coating materials and the two types of bacteria.

	Test	CFU n (10^4^)		ZOI Mean in mm		MTT % Live Bacteria		Confocal Microscope % Live Bacteria	
Material		SA	PA	SA	PA	SA	PA	SA	PA
PLGA		28	83	0.00	0.00	2.60	1.58	94.10	93.73
MP		0	22	29.67	18.33	3.36	1.90	97.26	83.28
CH		30	39	0.00	8.33	2.47	2.71	63.79	40.70
MC		0	27	21	13.33	3.69	3.47	42.63	38.79
MEM		3	3	20	14.33	14.03	24.24	46.61	46.61
Uncoated		164	327	0.00	0.00	57.99	94.09	99.74	99.43

## Data Availability

The data supporting the reported results analyzed or generated during the study are available upon request from the corresponding author.
